# Effectiveness and Safety of Short Course Liposomal Amphotericin B (AmBisome) as First Line Treatment for Visceral Leishmaniasis in Bangladesh

**DOI:** 10.1371/journal.pntd.0003699

**Published:** 2015-04-02

**Authors:** Emiliano Lucero, Simon M. Collin, Sujit Gomes, Fatima Akter, Asaduzzam Asad, Asish Kumar Das, Koert Ritmeijer

**Affiliations:** 1 Institute of Tropical Medicine and International Health, Charité—Universitätsmedizin Berlin, Germany; 2 Centro de Estudios e Investigación de la Enfermedad de Chagas y Leishmaniasis—Universidad Nacional de Cordoba, Argentina; 3 School of Social & Community Medicine, University of Bristol, United Kingdom; 4 Médecins Sans Frontières, Fulbaria, Bangladesh; 5 Médecins Sans Frontières, Amsterdam, The Netherlands; Institute of Tropical Medicine, BELGIUM

## Abstract

**Background:**

Bangladesh is one of the endemic countries for Visceral Leishmaniasis (VL). Médecins Sans Frontières (MSF) ran a VL treatment clinic in the most endemic district (Fulbaria) between 2010 and 2013 using a semi-ambulatory regimen for primary VL of 15mg/kg Liposomal Amphotericin-B (AmBisome) in three equal doses of 5mg/kg. The main objective of this study was to analyze the effectiveness and safety of this regimen after a 12 month follow-up period by retrospective analysis of routinely collected program data. A secondary objective was to explore risk factors for relapse.

**Methods and Principal Findings:**

Our analysis included 1521 patients who were initially cured, of whom 1278 (84%) and 1179 (77.5%) were followed-up at 6 and 12 months, respectively. Cure rates at 6 and 12 months were 98.7% (1262/1278) and 96.4% (1137/1179), respectively. Most relapses (26/39) occurred between 6 and 12 months after treatment. Serious adverse events (SAE) were recorded for 7 patients (0.5%). Odds of relapse at 12 months were highest in the youngest and oldest age groups. There was some evidence that spleen size measured on discharge (one month after initiation of treatment) was associated with risk of relapse: OR=1.25 (95% CI 1.01 to 1.55) per cm below lower costal margin (P=0.04).

**Conclusions:**

Our study demonstrates that 15mg/kg AmBisome in three doses of 5mg/kg is an effective (>95% cure rate) and safe (<1% SAE) treatment for primary VL in Bangladesh. The majority of relapses occurred between 6 and 12 months, justifying the use of a longer follow-up period when feasible. Assessment of risk of relapse based on easily measured clinical parameters such as spleen size could be incorporated in VL treatment protocols in resource-poor settings where test-of-cure is not always feasible.

## Author Summary

Visceral Leishmaniasis (VL) is a parasitic disease which is endemic in more than 80 countries, although 90% of cases occur in India, Bangladesh, Sudan, South Sudan, Ethiopia and Brazil. Most treatments are complex, expensive and require long application periods. AmBisome is one of the newest treatments available, but evidence for its safety and effectiveness under routine program conditions in resource-poor endemic areas remains sparse. Médecins Sans Frontières (MSF) ran a VL clinic from 2010 until 2014 in Fulbaria District, Bangladesh. Our retrospective study was based on all available data from this clinic, comprising 1521 patients diagnosed with primary VL who were treated with AmBisome 15mg/kg in three equal doses of 5mg/kg. We found that this treatment was safe (less than 1% of patients experienced a severe adverse event) and effective (more than 95% of patients were cured with one treatment) after 12 months. The youngest and oldest patients, and patients with large spleen size at the end of treatment, were more likely to experience a relapse. More than half of the relapses occurred between 6 and 12 months after treatment, therefore we recommend that clinical trials and treatment protocols adopt a minimum 12-month follow-up period.

## Introduction

Visceral Leishmaniasis (VL), also known as Kala Azar, is a vector borne disease caused by parasites of the genus *Leishmania*, *L*. *donovani*—*L*. *infantum* complex, which are transmitted through the bite of an infected sand fly (mainly genus *Phlebotomus*, old world, and *Lutzomya*, new world), Leishmaniasis infection in humans presents as cutaneous, muco-cutaneous and visceral. The visceral form is fatal if untreated. VL progressively affects the immune system of the patient, and opportunistic infections are frequently the final cause of death [1,2]. VL is endemic in around 80 countries, with over 90% of cases found in India, Bangladesh, Sudan, South Sudan, Ethiopia and Brazil [3].

The Fulbaria district of Bangladesh reported an average annual VL incidence rate of 17.8 per 10,000 people between 2008 and 2013 [4]. This far exceeds the government’s goal of one reported case per 10,000 habitants by the year 2015, as set in the Bangladesh VL Elimination Program in 2005 [4]. Until 2010, VL was treated in Bangladesh with Sodium Stibogluconate (SSG) or Miltefosine. SSG is given by intramuscular (IM) injections daily for 28 days. This course of treatment is painful, toxic, cumbersome for patients, and costly for the health system. In 2008, the national protocol for VL treatment in Bangladesh shifted to first line treatment with oral Miltefosine for 28 days. Miltefosine has gastro-intestinal side effects, is teratogenic (requiring 5 months of effective contraception for women of child bearing age, continuing for 3 months post-treatment [5]), and is prone to poor adherence if not administered under direct observation [6]. In 2013, AmBisome single dose (10mg/kg) was adopted as first line treatment, and this regimen is currently being rolled out across the country.

Médecins Sans Frontières (MSF) has run a VL control project in Bangladesh since 2010 by agreement with the Ministry of Health (MoH). In May 2010, MSF introduced an innovative first line therapy for primary VL comprising 15mg/kg liposomal amphotericin B (AmBisome) intravenous infusion, divided into three doses of 5 mg/kg given over a 5 day period, with only one night of hospitalization. Such a regimen had shown good efficacy and safety in a small clinical trial in India [7]. MSF introduced this regimen because the Bangladesh national VL guideline [8] allowed its use as second line treatment, although its effectiveness and safety had not been evaluated in routine clinical practice in south Asia. It was considered at that time that introduction of other regimens, such as a single dose [9][10], would require further evidence from clinical trials.

The aim of this study was to explore the effectiveness and safety of a short course AmBisome regimen of 15 mg/kg for primary VL under routine program conditions in Bangladesh, and to investigate predictive factors for relapse, with a 12 month follow-up period to assess final outcomes. Clinical studies in VL typically use a follow-up period of 6 months to establish final cure [11]. However, there is some evidence that most relapses occur later than 6 months post-treatment [12] [13], and wetherefore adopted a 12 month follow-up to allow the assessment of relapse rates up to 12 months post-treatment.

## Materials and Methods

This retrospective analysis used routine patient data from the MSF VL clinic in Fulbaria, Bangladesh, collected from January 2010 to April 2014. We included all patients diagnosed with primary VL who received AmBisome 15 mg/kg in three separate doses (5mg/kg), and who were not referred to other institutions for treatment or follow-up. Relapse VL cases require treatment with a higher total dose of AmBisome, and were excluded from our analysis.

### VL Protocol

In accordance with the MoH protocol [8], primary VL was suspected in patients with fever >2 weeks duration, weight loss, splenomegaly or lymphadenopathy, and no history of previous VL. VL was confirmed by rK39 rapid diagnostic test (RDT) (IT-LEISH, Bio-Rad Laboratories, USA). The performance of rK39 RDTs in South Asia has been shown to be consistently very high [14–16]. In a setting with a 50% prevalence of VL among clinical suspects this results in a very high positive predictive value. The patient’s general clinical condition, height, weight, spleen size and hemoglobin level (checked with HemoCue, HemoCue AB, Sweden) were recorded on admission to the clinic and when the patient was assessed for discharge (one month after admission). Pregnancy testing was done on admission. As the prevalence of malaria in this part of Bangladesh is extremely low, screening for malaria was restricted to vulnerable groups: age <2 or > 60 years and pregnant women, and to suspected malaria cases: patients with acute high fever (>39°C) or low hemoglobin (<8 g/dL). Routine HIV testing was not conducted due to the very low prevalence of HIV infection in this population.

Patients with confirmed primary VL were treated with intravenous liposomal amphotericin B (AmBisome, Gilead Pharmaceuticals, Foster City, CA, USA) using a total dose of 15mg/kg divided into three individual doses of 5 mg/kg: at admission (day 0); 24 hours after this dose, and five days after the first dose. Patients were routinely hospitalized for one day, or longer if severely ill. Patients showing a good clinical response (feeling well, resolution of fever, regression of spleen, and improvement of Hb [11]) were discharged from treatment after an assessment at 1 month. If there had not been a good clinical response, the possibility of treatment failure was checked by test-of-cure. Test-of-cure was by microscopy of Giemsa-stained smear of splenic aspirate. Patients who had missed any of the three doses were registered as “defaulted”. No parasitological test of cure was done routinely for primary VL cases. Patients presenting with VL relapse were treated with AmBisome (3 doses of 15mg/kg if their primary VL had not been treated with AmBisome, 5 doses of 25mg/kg if previously treated with AmBisome) followed by a test-of-cure after 28 days. Clinical presentation of relapse VL tends to be less severe but, in non-HIV-infected relapse patients, signs and symptoms remain typical (prolonged undulating fever, splenomegaly, anemia, weight loss). Diagnosis of VL relapse was confirmed parasitologically (by microscopy of Giemsa-stained smear of splenic aspirate).

### Follow-up

All patients were invited to attend a clinical follow-up appointment at 6 and 12 months after treatment or if ill health occurred. At each follow-up appointment a general clinical examination was done, including recording of temperature, spleen size and hemoglobin level. Outcome at each visit was recorded as: clinically cured (no signs or symptoms of systemic disease); sick but not VL (patients that were sick at the moment of the follow-up, but did not present signs and symptoms or clinical history suggestive of VL relapse); relapse (all patients with signs and symptoms of VL, confirmed by spleen aspirate microscopy if there were no contra-indications for splenic aspiration: spleen size >3cm below left costal margin, signs of active bleeding, increased bleeding and clotting time, severe anemia, jaundice, pregnancy, patient unable to remain still); or death. If not attending for follow-up, community health workers actively searched for the patient; if unsuccessful, the patient was classified as "lost to follow-up". Deaths during the follow-up period were determined by health staff using verbal autopsy to gather information on the cause of death and whether it could be related to VL. Molecular methods were not available for typing *Leishmania* strains in order to differentiate relapse from re-infection. PKDL was systematically looked for during follow-up, but was not considered a VL treatment failure.

### Statistical analysis

For the measurement of effectiveness we classified treatment outcome at one month as ‘initial cure’ for patients whose fever had subsided and who presented with spleen regression, increased hemoglobin level and weight, and improved general condition. Treatment outcomes at 6 and 12 months were categorized as: ‘cured’ (patients without sign and symptoms of VL); and ‘treatment failure’ (patients who had symptomatic VL relapse or who had died). For the measurement of safety we analyzed the frequencies of reported clinical complications that could be caused by VL treatment (vomiting, bleeding, and other). Severe adverse events (SAE) were defined as events leading to suspension or the cessation of VL treatment. Potential determinants of relapse for which there was evidence of an association in univariate analysis were carried forward to a multivariable logistic regression model that was adjusted (as *a priori* confounders) for age and sex. Predictive factors measured at discharge (one month after initiation of treatment) were adjusted for their values at baseline (admission) and all variables were added one by one to the final model. Continuous variables measured in adults *versus* children, and on admission *versus* discharge, were compared using Student’s t test. Data were analyzed with SPSS (SPSS Inc. Released 2008. SPSS Statistics for Windows, Version 17.0. Chicago: SPSS Inc.) and Stata (StataCorp. 2013. Stata Statistical Software: Release 13. College Station, TX: StataCorp LP)

### Ethical approval

This analysis met the Médecins Sans Frontières International Ethics Review Committee criteria for a study involving the analysis of routinely collected program data. The program utilized a recognized treatment for VL in Bangladesh, and was run in coordination with the Bangladesh Ministry of Health and Family Welfare through a memorandum of understanding, which is the usual procedure for NGOs operating in this context. All electronic data were analyzed anonymously.

## Results

**Fig 1:**
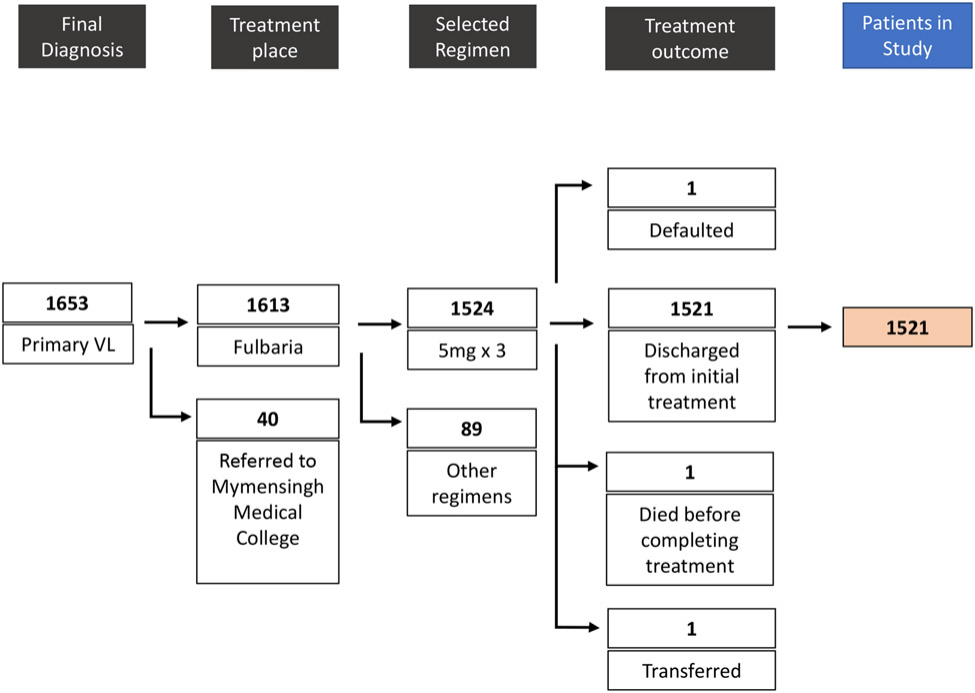
Patients included in this study.

After applying the inclusion criteria, a total of 1521 patients who completed the treatment were included in our study (Fig 1). The first and last admissions occurred on May 17^th^ 2010 and January 12^th^ 2013, respectively. The last patient to complete their 12 month follow-up attended the clinic on February 10^th^ 2014. Of the 1653 patients diagnosed with primary VL during this period, 40 complicated cases were transferred to tertiary care at Mymensingh Medical College. Of the 1613 patients treated at Fulbaria during the study period, 89 received other regimens: 18 patients with 5 x 3mg/kg had (suspected) impaired renal function and were treated with a lower dose more frequently over a longer period in accordance with protocol; 61 patients were treated with a single 10mg/kg dose as a new national protocol began to be introduced in 2013; and 10 patients were treated outside protocol for reasons which were not recorded in the database. The proportions of patients whose status was known at 6 and 12 months follow-up were 84.0% (1278/1521) and 77.5% (1179/1521), respectively. Data with which to analyze factors associated with risk of relapse at 12 months were available for 1106 patients.

### Baseline characteristics

The population was young (58.2% were <18 years old) and had similar age distributions in both sexes. The male:female ratio was approximately 1:1 across all age groups except among patients age 40+ years (3:1). The study population appeared to be under-nourished relative to WHO standards, with a mean BMI of 17.5kg/m^2^ for patients age ≥18 years, and a mean weight-for-height Z-score of −1.8 for patients <121 cm tall (see Table 1). No differences in baseline characteristics were observed between patients from different geographical areas. Adult and pediatric patients presented with similar Hb levels and spleen sizes, and were analyzed as a single patient group.

**Table 1:** Characteristics of the patients (N = 1521).

Age (years)	<5 n (%)	5–17 n (%)	18–39 n (%)	40+ n (%)	Total n (%)
	182 (12.0%)	702 (46.2%)	440 (28.8%)	197 (13.0%)	1521 (100%)
**Sex**					
Male	79 (43.3%)	393 (55.8%)	238 (54.1%)	151 (76.6%)	855 (56.5%)
Female	103 (56.6%)	310 (44.2%)	202 (45.9%)	46 (23.4%)	657 (43.5%)
Male:Female ratio	0.8	1.3	1.2	3.3	1.3
**Residence**					
Fulbaria	125 (68.7%)	523(74.5%)	302 (68.6%)	125 (63.5%)	1075 (70.7%)
Muktagachha	32 (17.6%)	69 (9.8%)	35 (8.0%)	25 (12.7%)	161 (10.6%)
Trishal	14 (7.7%)	54 (7.7%)	37 (8.4%)	16 (8.1%)	121 (8.0%)
Distant sub-districts*	11 (6.0%)	56 (8.0%)	66 (15.0%)	31 (15.7%)	164 (10.8%)
Anthropometric and clinical measures**	**n, mean (SD)**	**n, mean (SD)**	**n, mean (SD)**	**n, mean (SD)**	**n, mean (SD)**
Age (years)	182, 3.0 (1.0)	702, 9.7 (3.3)	440, 26.2 (5.6)	197, 49.4 (8.7)	1521, 18.8 (15.1)
Spleen size (cm) ^†^	181, 3.9 (2.1)	698, 4.8 (3.2)	439, 4.9 (3.9)	194, 3.8 (2.9)	1512, 4.6 (3.3)
Hemoglobin (g/dl)	174, 7.7 (1.5)	681, 8.8 (1.4)	428, 9.5 (2.0)	192, 9.7 (1.8)	1475, 9.0 (1.8)
Weight (kg)	182, 10.3 (2.1)	702, 23.3(9.9)	440, 42.3 (7.3)	197, 42.9 (7.5)	1501, 29.8 (14.2)
Height (cm)	178, 85.6 (9.4)	694, 126 (19.7)	437, 155 (8.6)	192, 156.7 (9.0)	1501, 133.6 (26.9)
BMI (over 18 years old)	-	-	395, 17.5 (2.2)	192, 17.4 (2.4)	587, 17.5 (2.2)
Z-score (weight-for-height up to 121cm tall)	173, −1.6 (1.2)	301, −2.0 (1.0)	-	-	474, −1.8 (1.1)

* Distant sub-districts (>20km from Fulbaria) include: Borishal, Dhaka, Gazipur, Jamalpur, Manikgonj, Mymensingh, Netrogona, Sherpur, and Tangail

** measured on admission

^†^ cm below lower costal margin

### Safety

During the treatment period, complications were registered as follows: bleeding 3.4% (52/1521); vomiting 9.5% (144/1521); and other complications (including fever, diarrheas, abdominal pain and local rash at the site of the injection) 0.4% (6/1521). Serious adverse events (SAE) were recorded for 7 patients (0.5%). The remainder had no SAE data, because there was no zero-reporting of adverse events. We cannot analyze the specific details of the SAE because no extra information was recorded in patient files. One of the SAE patients died before attending their first follow-up; of the remainder, none relapsed after 12 months of follow-up.

### Effectiveness

Improvements in key clinical parameters were noted at the one month follow-up: mean Hb levels increased from 8.99 to 10.87 g/dL (mean difference 1.90 g/dL (95% CI 1.80 to 1.95), P<0.001); mean spleen size decreased from 4.6 to 0.5 cm (mean difference −4.0 cm (95% −3. 9 to −4.2), P<0.001); and mean weight increased from 29.7 to 31.3 kg (mean difference 1.5 kg (95% CI 1.4 to 1.6), P<0.001).

Cure rates at 6 and 12 months were 98.7% and 96.4%, respectively (Table 2). A ‘best case scenario’ sensitivity analysis, in which we coded all patients lost to follow-up as ‘cured’ (16% (243/1,521) at 6 months, 22.5% (342/1,521) at 12 months), yielded a 6 month cure rate of 98.9% and a 12 month cure rate of 97.2%.

**Table 2:** Effectiveness analysis among patients discharged from treatment.

	6 month follow-up		12 month follow-up	
Excluding patients lost to follow-up	n/total	%	n/total	%
Failure (relapsed or died)	16/1278	1.3	42/1179	3.6
Relapsed	13/1278	1.1	39/1179	3.3
Died	3/1278	0.2	3/1179	0.3
Cured	1262/1278	98.7	1137/1179	96.4
Coding patients lost to follow-up as cured				
Failure (relapsed or died)	16/1521	1.0	42/1521	2.8
Relapsed	13/1521	0.8	39/1521	2.6
Died	3/1521	0.2	3/1521	0.2
Cured^†^	1505/1521	98.9	1479/1521	97.2

^†^ Numerator includes 243 patients lost to follow-up at 6 months and 342 patients lost to follow-up at 12 months

At 6 months follow-up, a total of 13 patients were diagnosed as relapse and 3 deaths were registered. After completing the 12 month follow-up, a total of 39 patients were diagnosed as relapse (including the previous 13 cases): 29 with parasitological confirmation; 10 without (because splenic aspiration was contra-indicated). Of these 10, none presented with lymphadenopathy. Local staff were not trained to perform bone marrow aspirates hence, lymph or bone marrow aspirates were not taken. No further deaths were registered.

Patients followed-up at 12 months did not differ in age, sex and clinical measures on admission and at discharge compared to patients not lost to follow-up, but patients lost to follow-up were more likely to reside in further away sub-districts, which we classified as “distant sub-districts” (27.5% *versus* 5.9%).

The three deaths occurred among male patients age >45 years: one entered treatment with a diagnosis of complicated advanced tuberculosis that was probably the main cause of death (after completing VL treatment he remained as an inpatient and died before the first follow-up); one died between the second and third follow-up from social violence (according to information provided by family members); and one suffered a severe adverse event (registered as acute jaundice) after he completed treatment and died at home before the first follow-up. This was the only case where the death could probably be attributed to VL and/or its treatment.

### Predictive factors for relapse

In Table 3 we summarize the results of a logistic regression model with relapse at 12 months as the outcome. We included only those patients with known status at 12 months (cured or relapsed) and who had complete data for potential risk factors (N = 1106). Odds of relapse were highest in the youngest and oldest age groups. There was no association with sex. Anthropometric indices for nutritional status (BMI in adults, weight-for-height Z-score in children under 121cm) were not associated with risk of relapse: for adults (n = 463), OR = 0.87 (95% CI 0.63 to 1.20) per kg/m^2^, P = 0.41; for children (n = 475), OR = 0.97 (95% CI 0.65 to 1.44) per weight-for-height Z-score, P = 0.88. Larger spleen size at discharge was associated with increased risk of relapse: OR = 1.25 (95% CI 1.01 to 1.55) per cm below lower costal margin, P = 0.04). We found tentative evidence of interaction (Likelihood ratio test P = 0.08) between discharge measurements of Hb and spleen size, which suggested an amplified combined effect of low Hb and large spleen size (S1 Fig).

**Table 3:** Factors associated with VL relapse at 12 months after treatment (N = 1106).

Risk factor	Odds ratio (95% CI) adjusted for age and sex	P-value	Odds ratio (95% CI) adjusted for all variables in table.	P-value
Sex				
Female	1.00 (reference)		1.00 (reference)	
Male	0.95 (0.47, 1.89)	0.88	0.75 (0.37, 1.52)	0.42
Age groups				
<5	9.72 (2.69, 35.1)	0.001	8.09 (2.03, 32.2)	0.003
5–17	2.69 (0.77, 9.44)	0.12	2.85 (0.78, 10.5)	0.12
18–39	1.00 (reference)		1.00 (reference)	
40 +	5.08 (1.29, 20.1)	0.02	6.19 (1.44, 26.6)	0.01
Admission Hb (per g/dL)	0.73 (0.58, 0.92)	0.006	0.83 (0.63, 1.09)	0.18
Discharge^†^ Hb (per g/dL)	0.69 (0.54, 0.87)	0.002	0.83 (0.62, 1.12)	0.23
Spleen size at admission (per cm below lower costal margin)	1.08 (0.98, 1.20)	0.13	0.96 (0.84, 1.10)	0.57
Spleen size at discharge^†^ (per cm below lower costal margin)	1.27 (1.10, 1.47)	0.001	1.25 (1.01, 1.55)	0.04

^†^ One month after admission (initiation of treatment)

## Discussion

Our study has shown that 15mg/kg AmBisome in three doses of 5mg/kg is an effective (96.4% cure rate) and safe (<1% SAE) treatment for primary VL in Bangladesh, when judged against internationally accepted parameters for effectiveness ≥95% and safety (SAE<5%) for VL treatment [1]. We demonstrated that VL treatment studies in this setting require a follow-up period longer than 6 months if they are to capture the majority of relapses. Whether routine follow-up of discharged patients for this length of time is necessary depends on ease of access to re-treatment for patients who relapse. In settings where access to re-treatment is problematic, routine follow-up may be equally difficult (and costly), but it could help to achieve the VL elimination target (of <1 case per 10,000 people at upazila level in Bangladesh) set in the Regional Strategic Framework for Elimination of Kala-azar from South East Asia Region [17]. We have also shown that residual splenomegaly is predictive of an increased risk of VL relapse, particularly in conjunction with low levels of hemoglobin.

The main strengths of our study are its size (>1500 patients), inclusivity (92% of primary VL cases during the period of the study), and extended follow-up (12-month). The main limitation of any cohort study is loss to follow-up. These were low (23% at 12 months) for a study of this design in a resource-poor setting, in part because outreach workers were employed to seek patients who did not attend follow-up appointments. A ‘best case scenario’ analysis showed a maximum cure rate of 97.2%. Although we cannot be certain about the validity of this scenario, ease of access to VL treatment in a relatively stable (non-migratory) population with multiple means of communication and transport (and given that apart from distance from facility, we found no differences between patients who were/were not lost to follow-up) suggest that the true cure rate is unlikely to be lower than 95%. Another limitation was the completeness of SAE reporting because non-occurrence of events was not recorded. Therefore we cannot verify that blank SAE values truly correspond to “treatment complete without SAE”. Although not all milder adverse events will have been reported and we are confident that no severe adverse events were missed, this is a limitation which we would seek to address in future studies by implementing a zero-reporting protocol for SAE.

AmBisome has been used to treat VL for more than 10 years [18], on the basis of evidence from clinical trials. However, evidence for the safety and effectiveness of AmBisome-based regimens under routine program conditions in endemic resource-poor areas remains sparse [19]. This has mainly been due to the high cost of the drug and the neglected status of the disease, factors which MSF has worked to address for many years [20]. In the meantime, studies based on routine data collection from VL treatment programs are an important source of evidence.

Our 6-month cure rate (98.7%) is consistent with rates reported from other studies of AmBisome [21], including several conducted in Bihar, India (a neighboring endemic region): Sinha *et al* reported 98.8% with 20mg/Kg in 4 doses over 10 days [22]; Sundar *et al* reported 95.7% with 10mg/Kg in single doses [9], 98.4% with a single 15mg/Kg dose [23], and 97.5% with combined doses of Liposomal Amphotericin B and Miltefosine [24]. In Bangladesh, in the neighboring sub-district of Muktagacha, Mondal *et al* reported a cure rate of 98% with a single dose 10mg/kg [25].

Our study provides further evidence that the standard 6 month follow-up period for VL studies or treatment programs [1] is insufficient. Several other studies have shown that most relapses occur after 6 months [13] [12] [26], and we would recommend that clinical trials and treatment programs adopt a 12-month follow-up period if relapse rates are to be measured accurately. However, even this extended length of follow-up is arbitrary, and we cannot discount the possibility that some patients relapsed after 12 months [13].

VL is associated with progressive weight loss and poor nutritional status. Conversely, malnutrition results in impaired immunity and is an important risk factor for severity of clinical VL [27] [28]. The very low anthropometric indices which we found in our patients are consistent with other VL patient cohorts in this population [25]. Whether these low indices are entirely a consequence of VL or whether they also indicate that chronic under-nutrition is a factor in perpetuating the endemicity of VL warrants further research.

That spleen size on discharge is a risk factor for VL relapse is consistent with findings from India [13] and South Sudan [29]. We also found tentative evidence of an amplified combined effect of low Hb and large spleen size, which is entirely plausible given that low Hb can be indicative of severity of VL disease (effect on bone marrow), and a massive spleen traps red cells and further depresses Hb [30–32]. Assessment of risk of relapse based on these easily-measured clinical parameters could be incorporated in VL treatment protocols in resource-poor settings where test-of-cure procedures cannot be routinely implemented, with closer and/or extended monitoring of at-risk patients.

## Conclusions

Our study has shown that 15mg/kg AmBisome in three doses of 5mg/kg is a safe and effective treatment for primary VL in Bangladesh, and could be an alternative to the current first line regimen of single dose 10mg/kg AmBisome. We have also shown that a follow-up period of 12 months is required to capture the majority of VL relapse cases, and that VL relapse is predicted by low Hb and large spleen size at the end of treatment. Until more evidence is gathered, we would recommend that follow-up be extended to 12 month for all patients or, where this is not feasible, 12-month follow-up could be targeted at patients who present with large spleen at the end of the treatment.

## Supporting Information

S1 Fig Effects of Hb on probability of relapse at 12 months by spleen size at time of discharge (one month after initiation of treatment). (TIF)
