# Supplementary figures and images for: Effectiveness and Safety of Short Course Liposomal Amphotericin B (AmBisome) as First Line Treatment for Visceral Leishmaniasis in Bangladesh
Source: PLoS Negl Trop Dis. 2015 Apr 2;9(4):e0003699. doi: 10.1371/journal.pntd.0003699 (PMC4383421; doi:10.1371/journal.pntd.0003699)

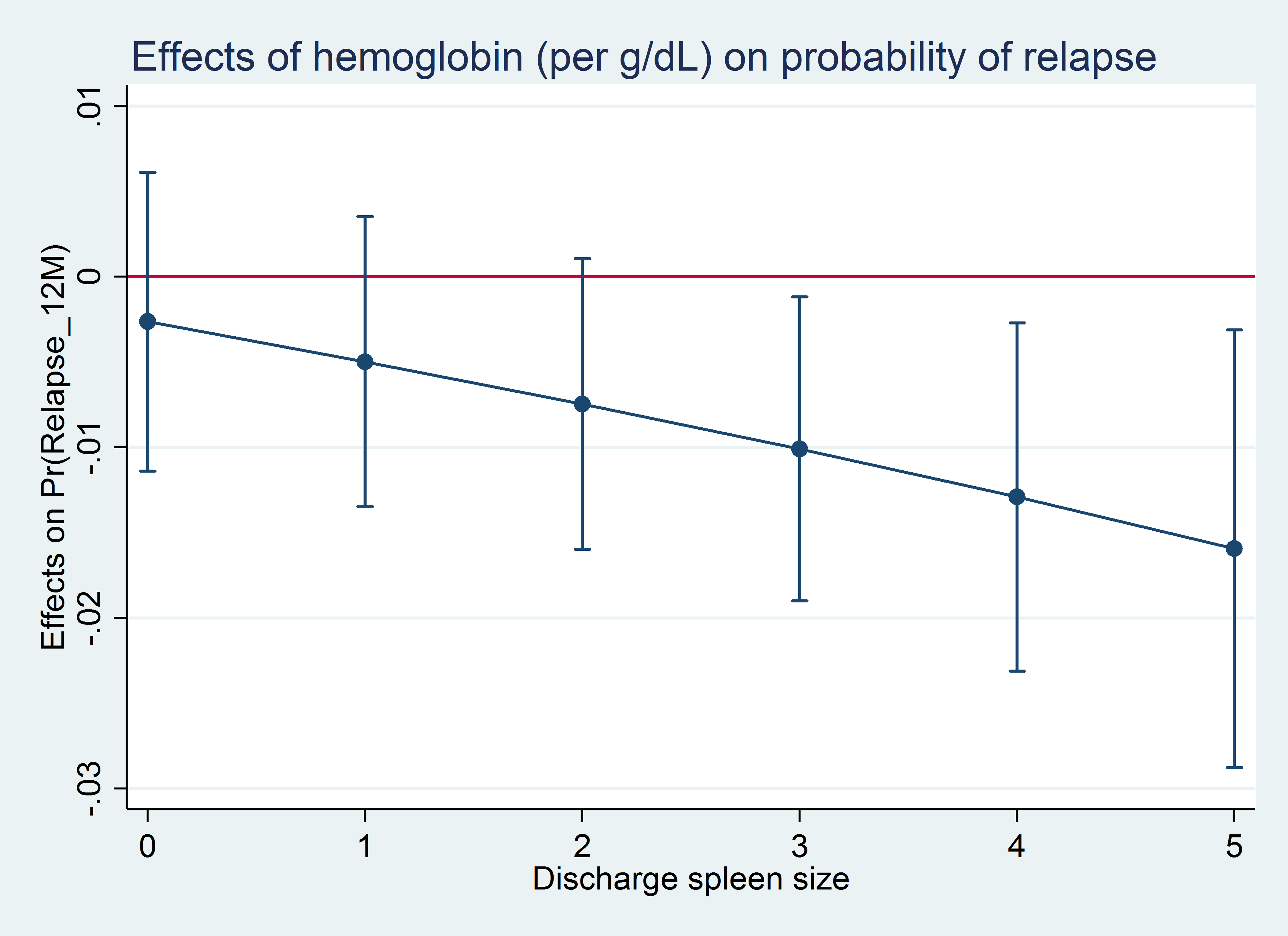

Supplement: S1 Fig — (TIF) [file pntd.0003699.s001.tif]
